# VisitSense: Sensing Place Visit Patterns from Ambient Radio on Smartphones for Targeted Mobile Ads in Shopping Malls

**DOI:** 10.3390/s150717274

**Published:** 2015-07-16

**Authors:** Byoungjip Kim, Seungwoo Kang, Jin-Young Ha, Junehwa Song

**Affiliations:** 1 School of Computing, Korea Advanced Institute of Science and Technology (KAIST), 291 Daehak-ro, Yuseong, Daejeon 305-338, Korea; E-Mails: bjkim@nclab.kaist.ac.kr (B.K.); junesong@nclab.kaist.ac.kr (J.S.); 2 School of Computer Science and Engineering, Korea University of Technology and Education (KOREATECH), 1600 Chungjeol-ro, Cheonan, Chungcheong 330-708, Korea; 3 Department of Computer and Communications Engineering, Kangwon National University (KNU), 1 Kangwondaehak-gil, Chuncheon, Gangwon 200-701, Korea; E-Mail: jyha@kangwon.ac.kr

**Keywords:** smartphone sensing, visit detection, place recognition, visit prediction, mobile advertising

## Abstract

In this paper, we introduce a novel smartphone framework called VisitSense that automatically detects and predicts a smartphone user’s place visits from ambient radio to enable behavioral targeting for mobile ads in large shopping malls. VisitSense enables mobile app developers to adopt visit-pattern-aware mobile advertising for shopping mall visitors in their apps. It also benefits mobile users by allowing them to receive highly relevant mobile ads that are aware of their place visit patterns in shopping malls. To achieve the goal, VisitSense employs accurate visit detection and prediction methods. For accurate visit detection, we develop a change-based detection method to take into consideration the stability change of ambient radio and the mobility change of users. It performs well in large shopping malls where ambient radio is quite noisy and causes existing algorithms to easily fail. In addition, we proposed a causality-based visit prediction model to capture the causality in the sequential visit patterns for effective prediction. We have developed a VisitSense prototype system, and a visit-pattern-aware mobile advertising application that is based on it. Furthermore, we deploy the system in the COEX Mall, one of the largest shopping malls in Korea, and conduct diverse experiments to show the effectiveness of VisitSense.

## 1. Introduction

In modern cities, large shopping malls such as the Mall of America and the COEX Mall [1] are places where people gather for shopping and leisure. Those places are usually large, crowded, and packed with a number of stores and restaurants. A large number of people gather at shopping malls, eat out, do shopping, or spend their leisure time. Accordingly, there exist many business opportunities, such as mobile advertising that targets the people who visit shopping malls and spend time and money there. The proliferation of smartphones opens up many new opportunities for mobile advertising in shopping malls. Many researchers expect that mobile advertising will be not only a killer application in mobile commerce but also an important business model for many emerging mobile applications to monetize [2,3,4]. Considering that explosive growth of the smartphone ecosystem has been mainly driven by a large number of ad-supported free apps in the app market, it is highly necessary to investigate how we can effectively provide mobile ads while satisfying both users and advertisers.

One of the most representative approaches to mobile advertising is location-based advertising (LBA). It provides nearby stores’ ads to mobile phones by sensing the users’ current location. For example, Alto *et al.* [5] proposed a system called B-MAD (Bluetooth Mobile Advertising) that sends ads to a mobile phone when a user passes by a certain stores. The proximity between a user and a store is detected by using a Bluetooth localization technique. However, LBA is highly limited in effective targeting. This is mainly because it delivers ads on nearby stores just depending on the user’s current location. For example, ads for nearby restaurants might not attract people who are having dinner or who recently ate.

In online advertising, *behavioral targeting* has been receiving significant attention as the most advanced targeting approach. Ads are presented based on an individual’s web-browsing behavior, such as the pages that they have visited or the searches they have conducted. The advantage of behavioral targeting comes from the fact that people who show similar behaviors, such as page view and search, will highly likely click the same ads on a page [6]. Such behavioral targeting can be applied to mobile advertising in shopping malls to increase the effectiveness of advertising. To enable behavioral targeting in the offline, physical world, however, it is not straightforward to accurately profile behaviors of customers (e.g., which stores or restaurants they visit, how much time they stay there, which places they are likely to visit, *etc.*) wandering around and spending time in shopping malls.

In this paper, we introduce a novel smartphone framework named VisitSense that automatically detects and predicts a smartphone user’s place visits via ambient radio to enable behavioral targeting for mobile ads in shopping malls. VisitSense enables mobile app developers to adopt visit-pattern-aware mobile advertising for shopping mall visitors in their apps. Using simple and intuitive VisitSense Application Programming Interfaces (APIs), the developers can harness the capability of being aware of smartphone users’ place visit patterns. Thus, they easily develop mobile apps powered by highly targeted mobile ads in shopping malls, which provide them with greater incentives. VisitSense is also beneficial to mobile users. It allows them to receive highly relevant mobile ads by being aware of the user’s place visit patterns in shopping malls. In addition, it allows users to have more comprehensive controls on their privacy since it processes and manages the privacy-sensitive sensor data on a smartphone without sending them to a server for data processing.

It is not trivial to automatically detect and predict the user’s place visits from ambient radio, such as Wi-Fi received signal strength indicator (RSSI) in shopping malls. There has been a research effort to detect place visits from ambient radio [7,8,9]. Their main idea is that ambient radio would be stable while a person visits a place, and a visit can be detected by tuning into such stable radio ambience. In many cases, however, the radio environment of the real world is very noisy; in such environments, existing algorithms easily fail. Furthermore, the problem is more challenging in shopping malls, since many customers often move around between shops and services that are in close proximity to each other. Moreover, predicting a place in which a user is likely to visit sooner or later is challenging since people’s behavior intrinsically has a high level of uncertainty.

To address the challenges, VisitSense employs a noise-robust visit place detection technique and a probabilistic prediction model. First, we propose a change-based visit detection method for accurately detecting the user’s place visits. Unlike the existing approaches, it attempts to detect changes in a transitional moment of entering and leaving a place. Detecting changes allows the method to better tolerate the noisy ambient radio. In addition, we adopt a noise-filtered Wi-Fi fingerprinting method to recognize a visit place. It filters out error-prone access points (APs) from Wi-Fi scans so that the fingerprints of adjacent places are more distinguishable from each other. Second, we propose a causality-based probabilistic prediction model to accurately predict the next visit places. Our basic idea is that we can reasonably predict a smartphone user’s next visits by learning people’s sequential visit patterns. For example, a dating couple who have visited a restaurant for dinner would highly likely visit a café or a bar for a drink rather than another restaurant. The proposed model can effectively exploit the causality that intrinsically exists in such sequential visit patterns.

The contributions of this paper are summarized as follows: First, we introduce a novel smartphone framework that automatically detects and predicts a smartphone user’s place visits for highly effective mobile advertising in shopping malls. Second, we identify problems with the existing visit detection algorithms in the noisy radio ambience environments, and develop a change-based visit detection method that is more tolerant to the noisy radio environments. We also develop noise-filtered Wi-Fi fingerprinting for accurately recognizing visit places. Third, we develop a causality-based probabilistic visit prediction model by using Bayesian networks, and show the feasibility of the model by using a dataset collected from about 130 participants in the COEX Mall, the largest shopping mall in South Korea. Finally, we build a VisitSense prototype system, and a visit-pattern-aware mobile advertising (VAA) application on top of it. Through experiments based on real deployment of the system in the COEX Mall, we show that VisitSense outperforms the existing visit detection algorithms. Furthermore, through a user study with 15 participants, we show that the VAA is more useful for users and it can be more cost-effective for advertisers than traditional LBA.

## 2. Related Work

### 2.1. Indoor Localization

Indoor localization has been an active research area, and a number of approaches for accurate and efficient localization have been presented [10,11,12,13,14,15,16]. Extensive effort has been made to develop Wi-Fi-based systems that utilize widely available Wi-Fi APs in an indoor environment. Wi-Fi-based indoor location systems build a radio map of an indoor environment in the training phase, and estimate a user’s most probable location from received Wi-Fi signals in the positioning phase.

Bahl *et al.* [11] proposed RADAR that uses an RSSI collected at multiple receiver points to triangulate the user’s coordinates. However, such deterministic approaches are very sensitive to the noise of Wi-Fi signal measurements. Accordingly, the triangulated location may have several meters of error, and may be inaccurate in tracking a user’s entrance or departure of a store-level place.

To increase the accuracy by effectively handling the noise of Wi-Fi signals, some researchers proposed probabilistic approaches. Zaruba *et al.* [12] suggested the use of Bayesian statistics to estimate a user’s location from RSSI in a home. They reduced the computational complexity of calculating posterior probability distribution by using Monte Carlo sampling.

In addition to Wi-Fi-based approaches, there have been other approaches including an active badge system that utilizes IR signal [10], a positioning system based on the magnetic signatures in the indoor environment [14], and a localization system based on FM radio signals [13]. A crowdsourcing approach has also been proposed to address the cost of building and maintaining Wi-Fi fingerprint databases for indoor localization by incorporating a number of user inputs. Park *et al.* addressed issues to determine when user input is required and to handle erroneous and stale data [15]. Chon *et al.* proposed a system to classify place categories (e.g., a store or restaurant) by collecting opportunistically captured images and audio clips from smartphones of users and utilizing unique characteristics of places in the data [16].

### 2.2. Place Detection and Recognition

Visiting a place can be detected by monitoring a person’s ambient radio. Due to its ubiquitous nature, radio fingerprints such as Wi-Fi fingerprints are usually applied as a fundamental technique. There are valuable research efforts that are being directed toward detecting place visits. Existing algorithms can be considered as stability-based visit detection. The stability-based approach considers that detecting a stable ambient radio indicates a stay at a place. The stability-based visit detection can be categorized into two methods: new-beacon-based and similarity-based algorithms. The new-beacon-based algorithms include BeaconPrint [7] and PlaceSense [8]. BeaconPrint detects an entrance if there are no new beacons (*i.e.*, APs) during a certain time window, and ascertains a departure if new beacons begin to appear in a time window. However, BeaconPrint is not robust to intermittent APs, since the intermittent APs cause false positives. To be more robust to intermittent APs, PlaceSense [8] uses a representative set of a place to avoid erroneously detecting a departure. A representative set of a place is a set of APs with high response rate during a stay at a place. PlaceSense detects a departure when all beacons of the representative set disappear or new beacons are found. The similarity-based algorithms include SensLoc [9]. SensLoc proposes using the similarity of Wi-Fi scans for accurately detecting a stable ambient radio. This technique is more robust to intermittent APs than the new-beacon-based one.

To detect the entrance and departure, some research uses both Wi-Fi and GPS signals. Morillo *et al.* [17] proposed an algorithm that detects an entrance by detecting the loss of GPS signals, and a departure by detecting the Wi-Fi fingerprints measured at the entry of a place. They developed such a hybrid entrance/departure detection algorithm to reduce the energy consumption of GPS on a mobile device. The algorithm is used to automatically turn off GPS when entering a building and to turn it on when departing from the building. However, the use of the algorithm is limited to only detect the moment when a user moves indoors or outdoors.

The use of radio fingerprints can easily fail to distinguish adjacent places, since radio signals are usually not confined to a certain place. To accurately recognize places, Azizyan *et al.* [18] proposed an accurate place recognition system called SurroundSense that uses diverse ambience fingerprints, such as sound, light, color, and user motion, in addition to radio fingerprints. However, the use of additional ambience fingerprints requires obtrusive and privacy sensitive ambience sensing, such as audio and video recording that may hamper the wide adoption.

### 2.3. Location Prediction

Recent research showed that people’s locations can be predicted with high precision since people show typical mobility patterns [19]. Location prediction can be broadly classified into two categories: geographic and semantic location prediction. Geographic location prediction provides geographic coordinates of the most probable location where a person will go next by learning a person’s transitions between her meaningful places. Ashbrook *et al.* [20] proposed using a Markov model to learn a person’s transition across some meaningful locations. Patternson *et al.* [21] proposed a method of learning a Bayesian model of a traveler moving through an urban environment. The model can learn the traveler’s current mode of transportation as well as his/her most likely route. Contrastingly, semantic location prediction provides semantic information, such as the category and activity of a place for a person to go next. Liao *et al.* [22] proposed relational Markov networks (RMNs) for predicting a person’s activities and significant locations from GPS trajectories. They showed that a person’s activities and locations can be recognized and predicted by location and time.

## 3. System Overview

VisitSense is a smartphone framework that detects and predicts a smartphone user’s place visits to enable behavioral targeting for mobile ads in large shopping malls. Continuously running on a smartphone, it monitors the changes within a smartphone user’s ambient radio and the user’s mobility to detect his/her place visits. If VisitSense detects an entrance or departure of a place, it sends corresponding events to applications so that they can exploit this information. More important, if VisitSense detects a departure from a place, it predicts places where it is highly likely that the user will. The prediction is valuable for being aware of the user’s potential visit places, which allows targeted mobile advertising.

### 3.1. System Architecture

**Figure 1:**
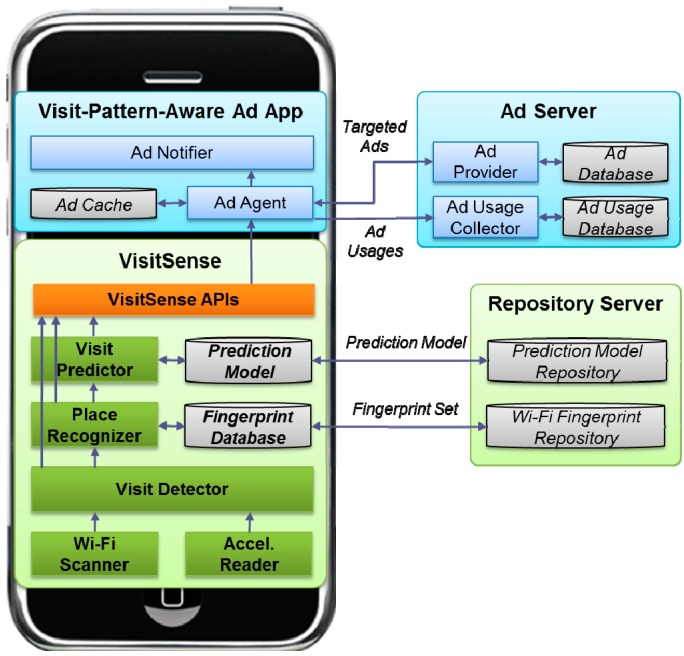
System architecture of VisitSense.

Figure 1 shows the system architecture of VisitSense. VisitSense consists of three main components: Visit Detector, Place Recognizer, and Visit Predictor. In addition to these, there are sensing components such as Wi-Fi Scanner and Accelerometer Reader. The Visit Detector is responsible for detecting a smartphone user’s entrance and departure from a place by monitoring Wi-Fi scans and accelerometer values. The Place Recognizer is responsible for recognizing the visit place by comparing the sensed Wi-Fi fingerprint to the predefined Wi-Fi fingerprints of the place. It maintains a Wi-Fi fingerprint database to match the fingerprints. The Wi-Fi fingerprint database for diverse shopping malls can be downloaded from a fingerprint repository. The Visit Predictor is responsible for predicting the user’s next visit place by evaluating the probability from the trained probabilistic prediction model. The trained model for diverse shopping malls can be downloaded from a model repository.

**Figure 2:**
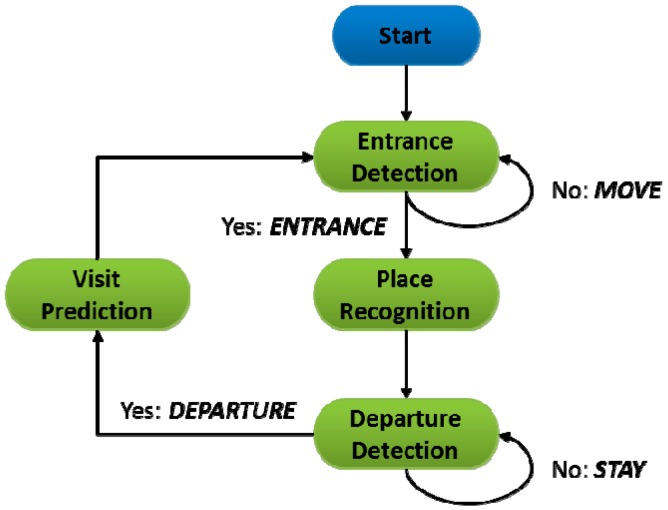
Overview of VisitSense operation.

Figure 2 shows the operation flow of VisitSense including visit detection, place recognition, and visit prediction. The visit detection includes both the entrance detection and departure detection.

### 3.2. Application Programming Interface

VisitSense provides app developers with two different types of APIs: Event and Query APIs. The Event APIs allow developers to receive events such as “entrance”, “departure” and “next visit” by subscribing to VisitSense. The Query APIs allow developers to access a smartphone user’s place visit history by querying VisitSense. These APIs are currently developed based on the Android platform.

**Figure 3:**
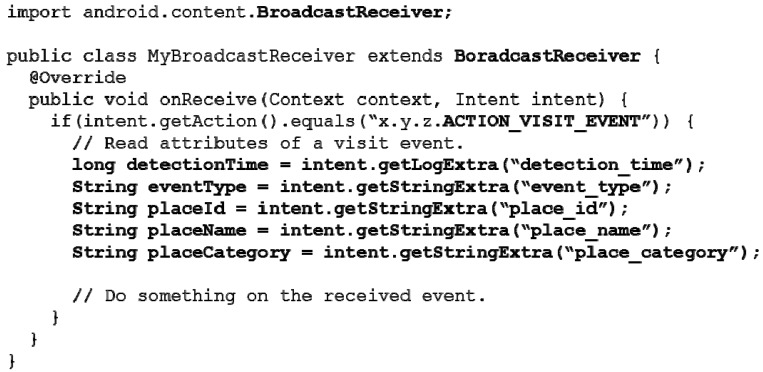
An example of using Event APIs of VisitSense.

Figure 3 shows an example of using the Event APIs. The Event APIs are implemented using the intent broadcasting mechanism of Android. By inheriting Broadcast Receiver, developers can receive events generated from VisitSense. The Query APIs are implemented using Content Provider of Android. Even though we introduce platform-dependent APIs, we believe that the proposed programming abstractions such as event and query can be implemented on other smartphone platforms (e.g., iOS).

### 3.3. Visit-Pattern-Aware Mobile Advertising

We implement visit-pattern-aware mobile advertising (VAA) on top of VisitSense. In our previous work, we introduced a visit-pattern-aware mobile advertising system named AdNext [3]. To send targeted ads, AdNext predicts a user’s next visit place, and sends some ads related to the predicted places. VisitSense has three main advantages over AdNext. First, VisitSense is a smartphone framework, and provides APIs that allow ad-supported mobile apps to be easily developed. In contrast, AdNext is simply a mobile advertising application that does not provide any APIs with which diverse mobile apps can be developed by providing mobile ads. Second, VisitSense provides better visit detection accuracy by developing several algorithms that are more tolerant of noisy ambient radio. Third, it allows users to have more comprehensive controls on their privacy, since it processes and manages privacy-sensitive data (e.g., visit places, entrance time, departure time, *etc.*) locally on smartphones. Instead of sending such privacy-sensitive data to the ad server, VisitSense pulls targeted ads from the ad server by predicting potential next-visit places. Unlike VisitSense, AdNext necessarily sends such privacy-sensitive data to a server, since the server analyzes the place visit history to select target ads.

## 4. Main Operations

In this section, we present the details of three main operations of VisitSense: visit detection, place recognition and visit prediction. Among three main operations, visit detection is a fundamental operation. This is because place recognition and visit prediction are triggered by visit detection. For each operation, we present the limitations of existing approaches and propose a new method.

### 4.1. Visit Detection

We briefly reviewed the existing visit detection algorithms in the Related Work section. The existing algorithms can be considered as stability-based visit detection. Their main idea is that ambient radio would be stable when a person visits a place, and a person’s visit can be detected by detecting a stable radio ambience. However, in the real world, the radio environment is very noisy.

#### 4.1.1. Challenge: Noisy Radio Ambience

We observed that existing visit detection algorithms easily fail in a real-world shopping mall such as COEX Mall [1]. We observed three challenging problems in the COEX Mall. We conjecture that the problems are caused by the characteristics of shopping malls: (1) places are closely allocated and (2) many APs are densely deployed. For example, in the COEX Mall, there are about 900 APs and, on average, about 22 APs are seen at each place. Note that, unlike VisitSense, all of the existing algorithms are evaluated in an environment where places are sparsely located (e.g., home, office, restaurant, *etc.*). Our observations are summarized as follows.

**Figure 4:**
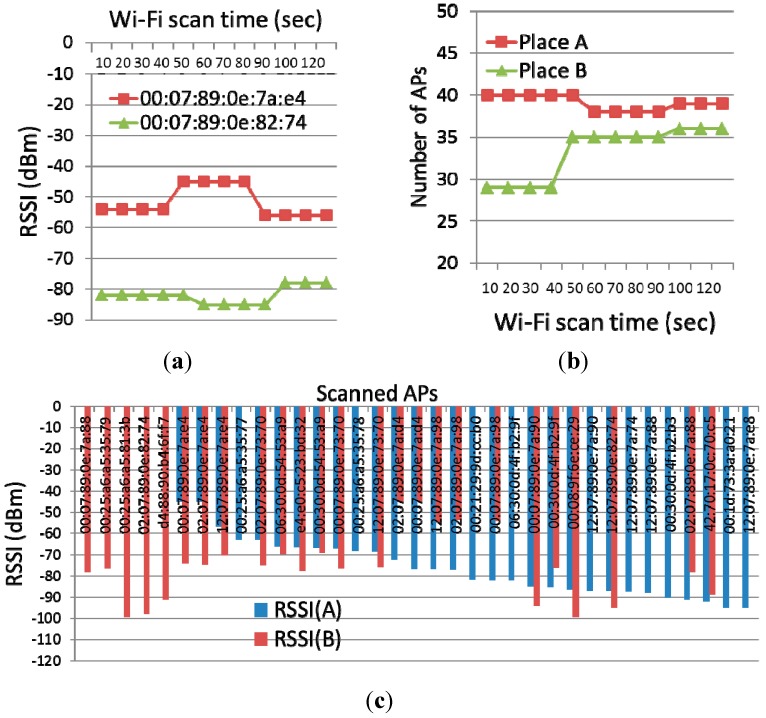
An example of (**a**) high RSSI fluctuation; (**b**) frequent AP churn; and (**c**) wide AP overlap.

**High fluctuations in the RSSI of Wi-Fi APs**: Figure 4a shows the changes in the RSSI of APs over continuous Wi-Fi scans at a certain place during a two-min period of time. Even at the same location, RSSI of APs highly fluctuates. The problem makes RSSI-based fingerprints error-prone in detecting a place. Therefore, for example, BeaconPrint [7] uses response-ratio-based fingerprints to recognize a place where a person enters.

**Frequent churn of Wi-Fi APs**: Figure 4b shows the changes in the number of APs over continuous Wi-Fi scans at a certain place during the course of two min. Even at the same place without moving, Wi-Fi APs frequently churn. More important, at place B, new APs suddenly appear. Note that the frequent churn incurs significant problems for the new-beacon-based algorithms, such as BeaconPrint [7] and PlaceSense [8]. This is because those algorithms mainly rely on the existence of new APs in the scan window for detecting entrance and departure.

**Figure 5:**
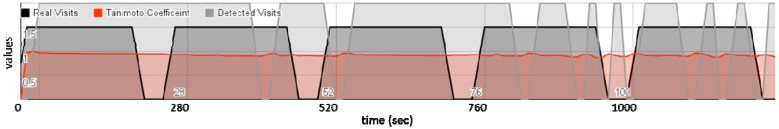
An example of the limitation of Wi-Fi scan similarity-based visit detection: Five adjacent places are visited. The black line represents real visits. The high values (*i.e.*, 1.5) of the black line indicate staying in a place, and the low values (*i.e.*, 0) indicate moving between places. The gray line represents the detected visits. The high values (*i.e.*, 2.0) of the gray line indicate staying in a place, and the low values (*i.e.*, 0) indicate moving between places. The red line represents the Wi-Fi scan similarity measured by the Tanimoto coefficient. The detected visits are determined by the comparison of the Tanimoto coefficient. The mismatch of the black line and gray line in horizontal axis means that there are many false positives and negatives for detecting the entrance and departure from a place.

**Wide overlap of Wi-Fi APs between adjacent places**: Figure 4c shows the distribution of APs between two adjacent places. The number of commonly scanned APs is 20. This is 80% of all APs seen at place B, and 67% at place A. This problem causes the similarity-based algorithms such as SensLoc [9] to fail. For example, in the COEX Mall, the Tanimoto coefficient (Table 1) is continuously high (*i.e.*, around 0.9) while moving around places. This problem is illustrated in Figure 5. This means that it is not a good measure to determine whether or not it is a visit. As a result, the similarity-based algorithms generate many false positives and negatives.

**Table 1:** Similarity measure algorithms used in VisitSense.

Algorithm	Formula
Jaccard coefficient	$J(F_{1},F_{2})=\frac{\left|F_{1}\cap F_{2}\right|}{\left|F_{1}\cup F_{2}\right|}$
Tanimoto coefficient	$T(F_{1},F_{2})=\frac{F_{1}•F_{2}}{{\left\|F_{1}\right\|}^{2}+{\left\|F_{2}\right\|}^{2}-F_{1}•F_{2}}$
Euclidean distance	$d(F_{1},F_{2})=\sqrt{\sum_{i=1}^{n}{\left(f_{1i}-f_{2i}\right)}^{2}}$
Pearson correlation coefficient	$r(F_{1},F_{2})=\frac{\mathrm{cov}(F_{1},F_{2})}{\sigma_{F_{1}}\sigma_{F_{2}}}$

#### 4.1.2. Change-Based Visit Detection

**Figure 6:**
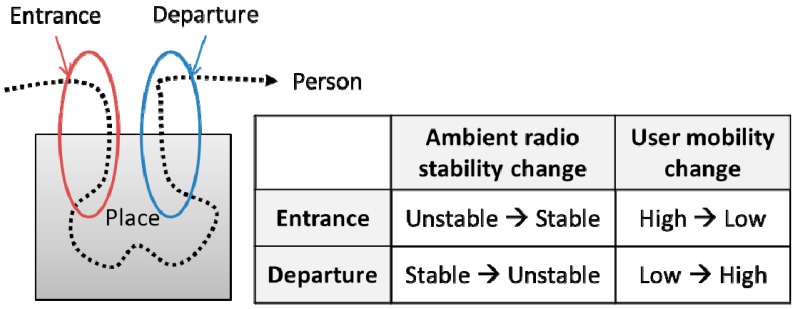
A concept of change-based visit detection.

For accurately detecting the entrance and departure in such challenging environments $a_{i,j}$ as those mentioned above, we propose a change-based visit detection method. The basic idea is that a transitional moment exists when a person enters or leaves a place. Then, the entrance and departure can be detected by detecting changes in such transitional moments. For example, when a person enters a place, the stability of the person’s ambient radio would become gradually stable, and the person’s mobility would become gradually lower. Reverse changes would occur when a person leaves a place. The basic idea is illustrated in Figure 6. The change-based visit detection is more robust to noisy radio ambience, since it attempts to detect the relative changes.

**Figure 7:**
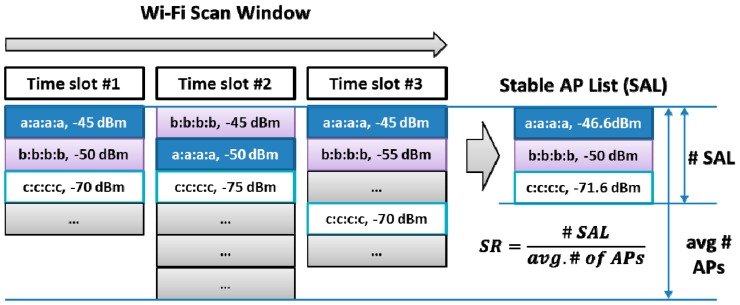
An example of a stable AP list (SAL).

VisitSense detects two changes: ambient radio stability change and user mobility change. First, to accurately detect an ambient radio stability change, we introduce a Stable AP List (SAL). A SAL is an ordered list of ambient APs that (1) continuously appears in a Wi-Fi scan window (e.g., response ratio: 1) and (2) shows strong RSSI (e.g., cutoff RSSI threshold: −90 dBm). A SAL is sorted in descending order of RSSI. The RSSI of SAL is an averaged value over a scan window. In Figure 7, an example of a SAL is illustrated.

To make the algorithm clear, we provide a simple formal notation. First of all, each scan at time *i*, $S_{i}$, can be represented as follow.

$$S_{i}=\left\{a_{i,j}|rssi(a_{i,j})>rssi(a_{i,j+1}),bssid(a_{i,j})\neq bssid(a_{i,k}),j=1,...,l\right\}$$

where $a_{i,j}$ is a *j*-th AP scanned at time *i*, $rssi(a_{i,j})$ is a RSSI value of $a_{i,j}$, $bssid(a_{i,j})$ is a BSSID (*i.e.*, the MAC address of the AP) value of $a_{i,j}$, and *l* is the number of elements of $S_{i}$.

Then, the SAL, $F_{t}$ can be represented as follows.

$$F_{t}=\left\{{\hat{a}}_{t,j}|rssi({\hat{a}}_{t,j})>rssi({\hat{a}}_{t,j+1}),bssid({\hat{a}}_{t,j})\neq bssid({\hat{a}}_{t,k}),rssi({\hat{a}}_{t,j})>\tau ,j=1,...,m\right\}$$

where ${\hat{a}}_{t,j}$ is a *j*-th element of a SAL generated at time *t*, $rssi({\hat{a}}_{t.j})$ is a RSSI value of ${\hat{a}}_{t,j}$, $bssid({\hat{a}}_{t,j})$ is a BSSID (*i.e.*, the MAC address of the AP) value of ${\hat{a}}_{t,j}$, τ is a cutoff RSSI threshold, and *m* is the number of elements of $F_{t}$. Here, $rssi({\hat{a}}_{t.j})$ is the average value of RSSI of the same AP in a scan window. More concretely, this is represented as follows.

$$rssi({\hat{a}}_{t,j})=\frac{1}{N}\sum_{u=0}^{N}rssi(a_{i+u,k}),\;here,\;bssid({\hat{a}}_{t,j})=bssid(a_{i+u,k})$$

Based on the concept of the SAL, for systematically detecting visits, we further define two fundamental measures: the *Stability Ratio (SR)* and *Change Ratio (CR)* of a SAL. The SR is a measure of how many stable APs exist in consecutive Wi-Fi scans in a time window. Accordingly, a high SR means that a user is highly likely to stay in a place. Formally, the SR can be represented as follows.

$$SR=\frac{n(F_{t})}{\frac{1}{N}\sum_{u=0}^{N}n(S_{i+u})}$$

where $n(F_{t})$ is the number of APs in a SAL $F_{t}$, and $n(S_{i+u})$ is the number of APs in a scan $S_{i+u}$. Here, t=i+N and N can be 2 for a window of three scans.

The CR is devised to represent the level of change in the SAL. More specifically, the CR will be a higher value if a more stable AP in the SAL shows changes in either the presence or the signal strength. Formally, CR can be represented as follows.

$$CR=\left\{\begin{array}{c} 1-a(j-1),if\left\{a_{t+u,k}|bssid({\hat{a}}_{t,j})=bssid(a_{t+u,k})\right\}=\text{∅}\\ 0.5-a(j-1),if\;rssi({\hat{a}}_{t,j})>rssi(a_{t+u,k})\wedge bssid({\hat{a}}_{t,j})=bssid(a_{t+u,k}) \end{array}\right\}$$

where α is a constant. The first condition of CR means that ${\hat{a}}_{t,j}$ (*i.e.*, top-*j* AP in a SAL $F_{t}$) disappears in the following scans. And, it means that if an AP with stronger RSSI disappears, CR will be higher. The second condition of the CR means that the RSSI of the ${\hat{a}}_{t,j}$ decreased in the following scans. For our experiments, we used 0.25 for α, and if ${\hat{a}}_{t,1}$ (*i.e.*, top-1 AP in a SAL $F_{t}$) disappears, the CR is 1. Since top-1 AP in a SAL is very stable and strong, the disappearance of this means a significant change in ambient radio.

**Figure 8:**
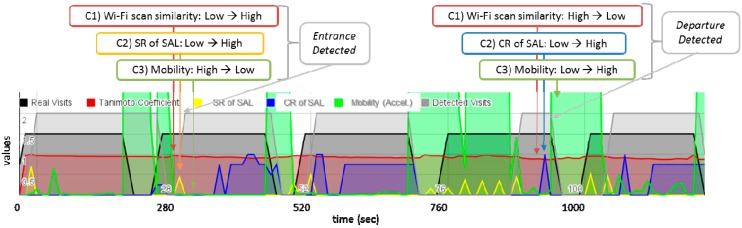
An example of visit detection of VisitSense. Five adjacent places were visited. The black line represents real visits. High values (*i.e.*, 1.5) of the black line indicates staying in a place, and low values (*i.e.*, 0) indicates moving between places. The gray line represents detected visits. High values (*i.e.*, 2.0) of the gray line indicates staying in a place, and low values (*i.e.*, 0) indicates moving between places. The red line represents the Wi-Fi scan similarity measured by the Tanimoto coefficient, and its values rage from 0 to 1. The yellow line represents the SR of a SAL, and its values ranges from 0 to 1. The blue line represents the CR of a SAL, and its values range from 0 to 1. The green line represents the mobility measured as the RMS of accelerometer values in a time window. This example shows that visiting five adjacent places were well detected by using the proposed algorithm. The details of algorithm are explained in the Section 4.1.2.

As mentioned earlier in this section, to detect entrance and departure, VisitSense monitors the ambient radio stability change and user mobility change. User mobility change is detected by accelerometer values. Using additional sensing modality, such as accelerometer effectively increases the detection accuracy by avoiding potential false positives due to erroneous changes of ambient radio. An example is provided in the following paragraphs (*i.e.*, Departure Detection) by using Figure 8. VisitSense uses a three-axies accelerometer on a smartphone. VisitSense uses the sum of the Root-Mean-Square (RMS) value of accelerometer values in a time window (e.g., sampling rate: 5 Hz, window size: 1 min) for each axis. If the value is greater than the threshold, it means high mobility. If not, it is low mobility. Accordingly, we can detect changes in mobility. This heuristic algorithm was developed since the goal is to detect a user’s movements that may lead to the entrance or departure of a place. A simple algorithm that detects whether an accelerometer value is greater than a threshold at a certain time may be considered. However, we could not use such an algorithm, since it may detect false positives, such as in a situation where a user uses his/her smartphone at the same location.

**Entrance Detection.** VisitSense detects the entrance to a place when the following entrance conditions are all fulfilled: (1) the similarity of Wi-Fi scans becomes high (e.g., *sim*(*S_1_*, *S_2_*) ≥ 0.9); (2) SR of a detected SAL becomes high (e.g., SR ≥ 0.4); and (3) a user’s mobility becomes low (e.g., *m* < 3). To show a clear example, we present a sensor log of VisitSense in Figure 8. Five adjacent places are visited, while staying at each place for about 200 s and moving to the adjacent place for 100 s. Each time a person enters places, a high SR is detected. In contrast, a low SNR is detected when a person is moving. Therefore, it can effectively avoid false positives.

**Departure Detection.** VisitSense detects the departure of a place when the following departure conditions are fulfilled: (1) the similarity of Wi-Fi scans becomes low (e.g., *sim*(*S_1_*, *S_2_*) < 0.9); (2) the CR of a detected SAL becomes high (e.g., CR > 0); and (3) a user’s mobility becomes high (e.g., *m* ≥ 3). Note that, unlike entrance detection, VisitSense checks whether either the first or the second condition is fulfilled. This is mainly because the similarity of Wi-Fi scans hardly changes while a user gradually moves around in the COEX Mall. In constant, the CR can immediately detect a user’s departure, since it is very sensitive to the change of Wi-Fi ambience. Note that such high sensitivity of CR is verified by user mobility. In Figure 8, we can see this case in the second visited place. Detecting even high CR is discarded since user mobility is still low. As shown in Figure 8, each time when a person leaves a place, the CR becomes high, while the Tanimoto coefficient is relatively constant.

### 4.2. Place Recognition

For recognizing a visited place, VisitSense uses Wi-Fi fingerprinting. To measure the similarity between Wi-Fi fingerprints, we can use some representative algorithms: the Jaccard coefficient, the Tanimoto coefficient, the Euclidean distance, and the Pearson correlation coefficient. These algorithms are briefly summarized in Table 1. The Jaccard coefficient is a measure defined by the number of the intersection divided by the number of the union of the two fingerprints (e.g., *F_1_* and *F_2_*). The Tanimoto coefficient is an extension for comparing vectors rather than sets. Euclidean distance is a measure of the distance between two Euclidean vectors in a Euclidean n-space. Finally, the Pearson correlation coefficient is a measure of the correlation (*i.e.*, linear dependence) between two fingerprints *F_1_* and *F_2_*, giving a value between +1 and −1 inclusive.

However, in the noisy Wi-Fi environment, Wi-Fi fingerprinting has problems: (1) Wi-Fi fingerprints generated at the same place may be considerably different from time to time and (2) Wi-Fi fingerprints generated from the adjacent places may be considerably similar.

#### Noise-Filtered Wi-Fi Fingerprinting

To address the problems mentioned above, VisitSense builds a Wi-Fi fingerprint database by using noise-filtered Wi-Fi fingerprints. A noise-filtered Wi-Fi fingerprint is made by filtering out error-prone APs so that they are more static over time and more distinguishable among each other. To achieve this goal, we used a simple heuristic of filtering out APs that (1) intermittently appear in a scan window and (2) have weak RSSI. The second heuristic comes from our observation that APs with weak RSSI highly fluctuates in their RSSI over time, compared to APs with strong RSSI. In other words, at a certain place, APs with weak RSSI usually show a larger standard deviation of RSSI than APs with a strong RSSI. This simple heuristic for filtering policy shows impressive improvements in recognition accuracy. The details are presented in the evaluation section.

VisitSense simply implements the noise-filtered Wi-Fi fingerprinting by using a SAL. After VisitSense detects an entrance to a place by detecting a SAL with a high SR, it uses the detected SAL as a noise-filtered Wi-Fi fingerprint to the visited place.

### 4.3. Visit Prediction

Generally, predicting a person’s next visit place is challenging, since people’s behavior intrinsically has a high level of uncertainty. However, at some level of granularity, it is predictable due to the nature of the repetitive patterns of people’s daily lives. Our basic idea is that we can reasonably predict smartphone user’s next visits by learning people’s sequential visit patterns. This intuition comes from the following two reasons. First, there are frequent and common visit patterns among many people. For example, we observed that many people visit the COEX Mall to have fun on weekends and usually visit a cinema, a restaurant, and a café. Second, visit causality exists in such visit patterns. For example, a person who has dinner now does not go to a restaurant again within the next few hours. Accordingly, we can predict a person will highly likely visit a café next if the person has already visited a cinema and a restaurant on the weekend at the COEX Mall.

#### Causality-Based Visit Prediction Model

To effectively capture the causality in the sequential visit patterns, we build a probabilistic model using Bayesian networks. A Bayesian network (BN) [23] is a probabilistic graphical model that represents a set of random variables and their conditional dependencies via a directed acyclic graph. A BN models the joint probability P(**C**, **F**), where **C** represents classes (or labels) and **F** represents features (or observations). To make inference tractable, BN assumes conditional independences among **C** and **F**, and represents the joint probability P(**C**, **F**) as products of conditional probabilities. It is represented by the following formula.

$$P(V)=\prod_{v\in V}P(v|\;parents(v))$$

where **V** represents nodes (or vortexes) in a Bayesian network (*i.e.*, including both **C** and **F**), and parents(*v*) represents parent nodes linked with a node *v* by edges.

We consider visit place, visit time, visit duration, gender and age as the main features for a prediction model. Visit place is denoted by **P**. **P** has four states, *i.e.*, shopping store, café, restaurant, and entertainment place. Visit time is denoted by **T**. **T** has 24 states which are divided by hour. Visit duration is denoted by **D**. **D** has eight states whose max value is limited to four hours and each state is divided into 30-min chunks. Age is denoted by **A**. **A** has four states, *i.e.*, under 20 s, 20 s, 30 s, and over 40 s. Gender is denoted by **G**, and has two states, *i.e.*, male and female. We further denote the current features by **P**_0_, **T**_0_, and **D**_0_, and the *i*-th previous features by **P**_i_, **T**_i_, and **D**_i_. Note that age **A** and gender **G** are static features.

**Figure 9:**
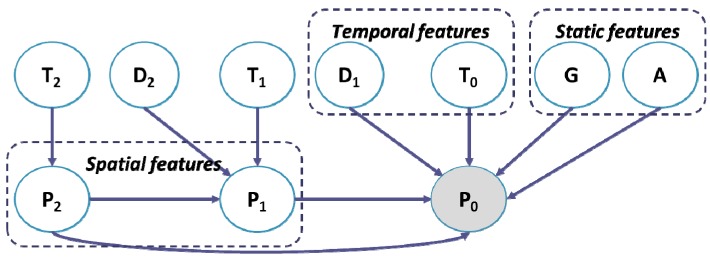
A Bayesian network for visit prediction.

Figure 9 shows a causality-based visit prediction model using Bayesian networks. Conceptually, in the model, an edge represents the causality between linked nodes (or random variables). More specifically, the model implies that current visit place **P**_0_ is influenced by special features such as the previous visit place **P**_1_ and **P**_2_, and temporal features such as the previous visit duration **D**_1_ and current time **T**_0_. In addition, it is influenced by profile features such as gender **G** and age **A**. The conditional probability distributions of the proposed BN have been learned by using the E-M algorithm [24].

## 5. Evaluation

For evaluating VisitSense and a visit-pattern-aware mobile advertising (VAA), we generated a Wi-Fi fingerprint database for a real-world shopping mall (*i.e.*, COEX Mall [1], the largest shopping mall in South Korea). Only one fingerprint was defined for each place (*i.e.*, in total for about 120 places). Then, we evaluated VisitSense using three different datasets collected in the COEX Mall. First, we collected a Wi-Fi scan log at different places to evaluate the place recognition accuracy. Second, we collected a sensing log including both Wi-Fi scans and accelerometer values to evaluate the visit detection accuracy. Third, we collected survey data consisting of a list of visited places from about 130 participants to evaluate the visit prediction accuracy. Further, we conducted a preliminary user study by recruiting 15 participants to evaluate the effectiveness of the visit-pattern-aware mobile advertising. For the evaluation, we used Android smartphones (*i.e.*, Motorola XT70 and HTC Desire). While the two smartphone models are limited to cover a number of phone models with different specifications, we believe that they suffice to examine the feasibility of our proposed methods and the effectiveness of VisitSense framework in the initial stage. More in-depth study to examine the performance variation of different smartphone models will be necessary in future work.

The main parameters of VisitSense are presented in Table 2. The default values are indicated by *. In the remaining section, we mention specific parameter settings only when the default values were not used.

**Table 2:** Parameters of VisitSense.

Operation	Parameter	Values (* = default)
Wi-Fi Scan Window	Wi-Fi scan period	10 *, 30 (s)
	Wi-Fi scan window size	3 *
Wi-Fi Ambience Change Detection	Tanimoto similarity threshold	0.9 *
	Cutoff RSSI threshold	−60, −90 *, −120 (dBm)
	SR of SAL	0.4 *
	Top-k AP RSSI change	25 dBm *
Mobility Change Detection	Accel. sampling frequency	5 Hz *
	Accel. window size	300 (1 minute) *
	Mobility threshold	3 *
Place Recognition	Similarity algorithm	Tanimoto coefficient *, Jarccard coefficient, Euclidean distance, Pearson correlation coefficient

### 5.1. Place Recognition Accuracy

#### 5.1.1. Data Collection

We collected a Wi-Fi scan log in the COEX Mall. Wi-Fi scans were collected every 10 s (*i.e.*, 1/10 Hz). To examine how well VisitSense distinguish adjacent places, we evaluated VisitSense at five adjacent places in three different sections (*i.e.*, Section M, Section F, and Section O) of the COEX Mall. That is, we collected Wi-Fi scans at 15 adjacent places. For correctness, collecting and evaluating were performed five times at each place.

#### 5.1.2. Evaluation Results

**Figure 10:**
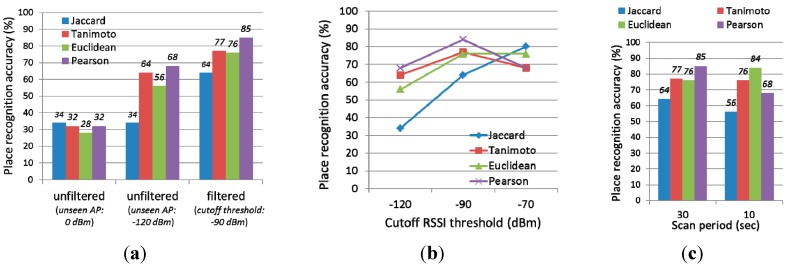
Place recognition accuracy. (**a**) w.r.t similarity algorithms; (**b**) w.r.t cutoff threshold; and (**c**) Wi-Fi scan period.

First, we evaluated the effect of using noise-filtered Wi-Fi fingerprinting on the place recognition accuracy. To make sure, we used four similarity measure algorithms. Figure 10a shows the evaluation results. When using noise-filtered Wi-Fi fingerprinting, the place recognition accuracy increased for all the similarity algorithms. For example, when using Pearson correlation coefficient, the accuracy is increased from 68% to 85%. Note that, when comparing two fingerprints with different APs, SensLoc set the RSSI of complemented APs to 0 dBm to make the fingerprints correspond. However, as shown in the figure, this resulted in poor accuracy. Therefore, in VisitSense, we used −120 dBm for complement APs. Using weak RSSI for complement APs is more reasonable.

Second, we evaluated the effect of the cutoff RSSI threshold on the place recognition accuracy. Figure 10b shows the evaluation results. The place recognition accuracy is the highest when the cutoff RSSI threshold is −90 dBm. Therefore, we can empirically conclude that −90 dBm is a reasonable value for the cutoff RSSI threshold of VisitSense. Note that, when using a high cutoff RSSI value (*i.e.*, −70 dBm), the accuracy adversely decreases. We assume that this is mainly because the Wi-Fi fingerprints are filtered too much. In contrast, when using the Jaccard coefficient, the accuracy still increases even when using high cutoff RSSI threshold. This is mainly because Jaccard coefficient only considers the ID of APs without RSSI.

Third, we evaluated the effect of different Wi-Fi scan frequencies on the place recognition accuracy. Figure 10c shows the evaluation results. When the Wi-Fi scan period is set to 30 s, the place recognition accuracy is slightly higher than 10 s. However, the difference was not significant.

### 5.2. Visit Detection Accuracy

#### 5.2.1. Data Collection

We collected sensor data traces including both Wi-Fi scans and accelerometer values. The sensor data traces were collected by performing a scenario-based tour of the COEX Mall. The tour was designed by considering a worst-case scenario, where a person would continuously visits adjacent places. More specifically, in one of the most popular sections (*i.e.*, Section F), a person would hypothetically visit five adjacent places, staying about 200 s (*i.e.*, 20-scan period) before moving to the next place for 100 s (*i.e.*, 10-scan period). Note that, in shopping malls, this worst-case scenario usually happens (e.g., shopping). We conducted five repeated tours for correctness of evaluation.

#### 5.2.2. Methodology

**Figure 11:**
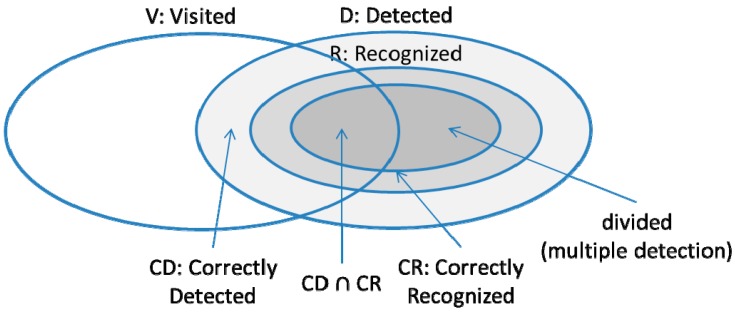
Visit detection evaluation methodology.

For comparison, we implemented SensLoc [9], a state-of-the-art visit detection system. For SensLoc, we set scan period, scan window size, certainty value, and Tanimoto threshold to 10 s, 3 s, 2 s, and 0.9 s, respectively. We measured the precision and recall of SensLoc and VisitSense. The precision and recall of visit detection are defined as follows. Here, *V* represents a set of ground-truth visits, *D* represents a set of visits detected by each system, *CD* represents a set of visits correctly detected, and *CR* represents a set of visits correctly recognized. To clear the discussion, we present the relationship of these sets in Figure 11. Conceptually, the visit detection accuracy means the accuracy of correctly detecting an entrance to a place and also correctly recognizing the visited places.

$$precision=\frac{\left|CD\cap CR\right|}{\left|D\right|},\;recall=\frac{\left|CD\cap CR\right|}{\left|V\right|}$$

For clear discussion, we differentiate visit detection accuracy from entrance detection accuracy and place recognition accuracy. Furthermore, we define the visit sequence detection accuracy as the accuracy of correctly detecting two consecutive visits. The visit sequence accuracy is important since the visit prediction operation is based on the two previous visit places for predicting the next visits. Note that, however, the visit prediction operation can work even if one of the previous visits has been incorrectly detected.

#### 5.2.3. Evaluation Results

**Figure 12:**
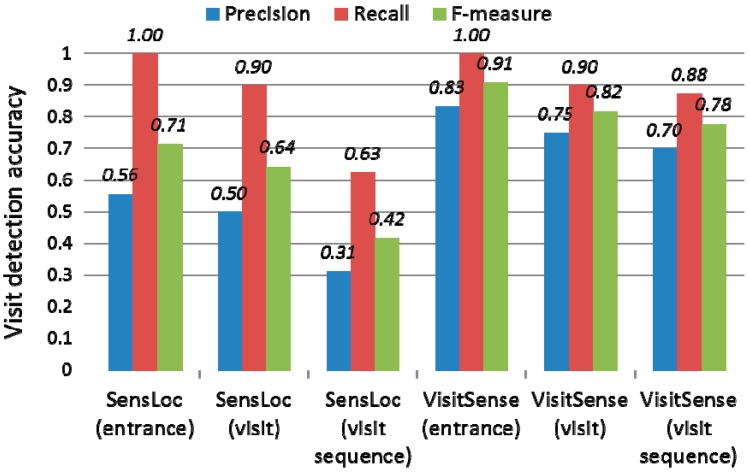
Visit detection accuracy comparison.

First, we evaluated the single visit detection accuracy. Figure 12 shows the evaluation results. The left three groups are the evaluation results from SensLoc, and the right three groups are from VisitSense. First of all, the entrance detection precision of VisitSense is 0.83, and the recall is 1. Next, the visit detection precision of VisitSense is 0.75, and the recall is 0.9. Note that, the precision of VisitSense increased from 0.75 up to 0.9 by applying the post processing that merges divided visits to the same place. However, to exclusively measure the benefit due to only the change-based visit detection, we do not include the benefit of the post processing. In contrast, the visit detection precision of SensLoc is only 0.5, and the recall is 0.9. Showing about 50% better performance, VisitSense outperforms SensLoc in the very noisy radio ambience. This is mainly because VisitSense uses means such as a stable AP list (SAL) and user mobility, which are more tolerant of the noisy radio ambience. In noisy radio ambience environments, only monitoring the similarity of radio scans easily fails in accurately detecting visits.

Second, we evaluated the visit sequence detection accuracy. Figure 12 shows the evaluation results. The visit sequence detection precision of VisitSense is 0.70, and the recall is 0.86. In contrast, the precision of SensLoc is only 0.31, and the recall is 0.63. The precision of VisitSense is almost two times better than that of SensLoc. Note that, the visit sequence detection precision of VisitSense slightly decreased from the single visit detection precision (*i.e.*, from 0.75 to 0.70). Unlike VisitSense, the precision of SensLoc considerably decreased (*i.e.*, form 0.50 to 0.31). This is mainly because VisitSense detects single visits more accurately than SensLoc in the noisy radio ambience and hardly generates erroneous visits between true visits.

### 5.3. Visit Prediction Accuracy

#### 5.3.1. Data Collection

We collected a visit history of COEX Mall users. We requested randomly-selected users to list all the places they visited. More specifically, we requested some people who entered COEX Mall to participate in our user study. For people who accepted our request, we asked them to write down visit time, visit duration, and their rating of the places visited. We surveyed about 130 people. From the data, we filtered out some users who reported too few visit places (*i.e.*, less than three places), or whose data were duplicate to others (*i.e.*, possibly companions such as couples or families). We thus obtained a clean dataset of 76 people. The demographics of these users are summarized in Table 3. The total number of visits was 351. We used 80% of the entire dataset as a training dataset and 20% as the test dataset (*i.e.*, 80% split).

**Table 3:** The user demographics of the COEX place visit dataset.

	Attribute	Number	Ratio
Sex	Man	22	29%
	Woman	52	68%
	N/A	2	3%
Age	~19	9	12%
	20–29	48	63%
	30–39	14	18%
	40~	2	3%
	N/A	3	4%
Job	student	35	46%
	employee	27	36%
	others	7	9%
	N/A	7	9%
Total		76	

#### 5.3.2. Evaluation Results

**Figure 13:**
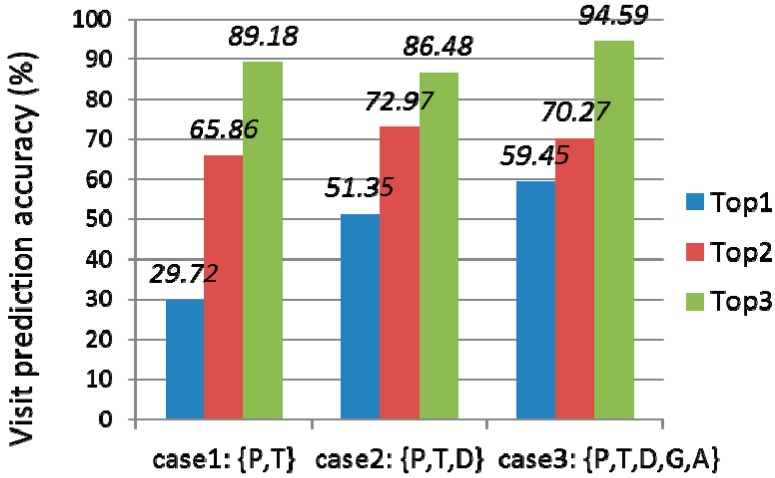
Visit prediction accuracy w.r.t features.

First, we evaluated how much the features of the model affected the visit prediction accuracy. We used four place categories, *i.e.*, shopping store, café, restaurant, and entertainment place. We trained the proposed BN using GeNIe and SMILE [25], a Bayesian network toolkit. Figure 13 shows the evaluation results. As the features of the model increase, the prediction accuracy increases. Case 1 only considers visit place **P** and visit time **T** as features. It is derived from the model in Figure 9 by eliminating gender **G**, age **A**, and visit duration **D**. Case 2 includes visit place **P**, visit time **T**, and visit duration **D**. Case 3 includes all the features. In the figure, top1 means the percentage accuracy that the next visit place is correctly predicted as the first rank (*i.e.*, the highest probability), and top2 means the percentage accuracy that the next visit place is correctly predicted within the second rank (*i.e.*, the second highest probability). Case 3 shows the highest prediction accuracy, *i.e.*, 59.45%. If top2 is considered, the prediction accuracy of the model increases to 70.27%. We believe the prediction accuracy would further increase as the number of training data increased.

Second, we compared the BN-based prediction model with other models. We used a decision tree for a non-probabilistic model, and conditional random fields (CRFs) for a competitive probabilistic model. For evaluating the diction tree (*i.e.*, C4.5), we used Weka [26], a data mining and machine learning toolkit. For evaluating CRFs, we used a Java CRF package [27]. We used default settings provided by the package. Table 4 shows the evaluation results. The decision tree showed about 40.54% accuracy. CRF shows about 29.72% accuracy. The evaluation shows that a BN-based model outperforms other models. When the independence assumption holds, or when only a small amount of training data is available, generative models such as BNs could outperform discriminative models such as CRFs [27].

**Table 4:** Visit prediction accuracy comparison.

Classifier		Evaluation Method	Prediction Accuracy (%)
	Structure Learning Algorithm		
Decision tree	N/A	80% split	40.54
CRFs	N/A	80% split	29.72
Bayesian networks	Domain knowledge	80% split	59.45
	Repeated Hill Climbing	80% split	65
		cross-validation	52.76
	Inferred Causation	80% split	55
		cross-validation	51.76

Third, we evaluated the effectiveness of the proposed BN structure (*i.e.*, a DAG of a BN) compared to automatically generated structures. There are various algorithms for learning structures of BNs. We used two representative algorithms: Hill Climbing [28] and Inferred Causation [29]. For the evaluation, we used Weka [26]. Table 4 shows the evaluation results. The BN generated by the Repeated Hill Climbing shows 65% accuracy, and the one generated by the Inferred Causation shows 55% accuracy. This shows that the proposed BN generated by domain knowledge performs as well as the BNs generated by algorithms. In other words, our visit causality assumption is empirically reasonable. For fair evaluation, we also measured the prediction accuracy using cross-validation (*i.e.*, 10-fold) which repeats evaluations over divided datasets to avoid the skew-ness of a training dataset.

### 5.4. Preliminary User Study

#### 5.4.1. Methodology

To evaluate the effectiveness of visit-pattern-aware mobile advertising (VAA), we conducted a preliminary user study at the COEX Mall. We measured the extent to which participants felt the received ads were useful for them. For comparison, we also built an experimental location-based advertising (LBA) system. Unlike VAA, LBA sends ads when a user walks into the vicinity of a place. For the evaluation, we selected 50 advertising places: 25 shopping stores, seven cafes, 14 restaurants, and four entertainment places. The number of places was chosen by considering the proportion of each place category. Then, we recruited 15 participants through help-wanted ads on the Internet. The demographics of the participants are summarized in Table 5. We requested the participants use both VAA and LBA for two hours on different weekends. In addition, we requested them to give a 5-scale rating for each ad they received.

**Table 5:** The user demographics of preliminary user study.

	Attribute	Number
Sex	Man	7
	Woman	8
Age	20–22	5
	23–26	8
	27–29	2
Affiliation	KAIST	8
	Others	7
Total		15

#### 5.4.2. Results

**Figure 14:**
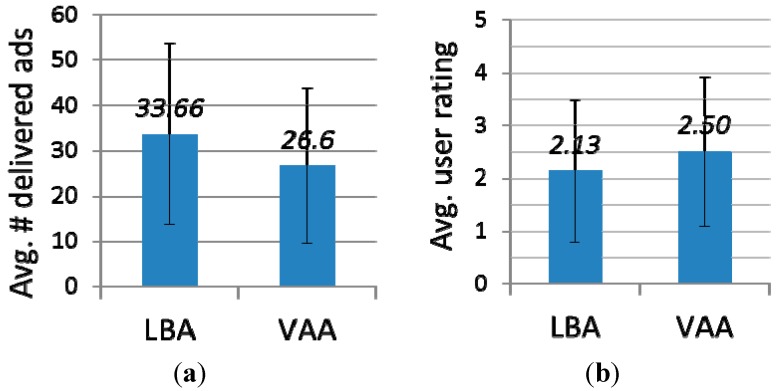
User study results (**a**) the average number of delivered ads; (**b**) the average rating of delivered ads.

Figure 14a shows the average number of ads each participant received during the experiment. Each participant received an average of 33.66 ads from LBA, and an average of 26.6 ads from VAA. That is, participants received about 26.3% more ads from LBA than VAA. This is mainly because in the case of VAA, participants receive ads only when they leave a place. Instead, in LBA, participants unnecessarily receive many ads when they simply pass by a place. Figure 14b shows the average rating the participants gave to each ad. The average rating was 2.13 for LBA, and 2.50 for VAA. This indicates participants thought VAA ads were more useful than LBA ads. Through this user study, we can empirically conclude that VAA can provide more useful ads for users while sending fewer ads. This means advertisers can increase the effectiveness of their campaign while reducing the cost to send ads.

## 6. Limitation and Discussion

**User acceptance:** It is important to provide actual benefits to users in order to make them use such applications for visit-pattern-aware mobile ads. We believe that many mobile users have been increasingly interested in mobile coupons or ads and will gain benefits from mobile ads or coupon apps. Mobile apps to provide coupons of offline stores have been increasingly emerging. According to an Accenture survey [30], 79% of smartphone users found it useful to download mobile coupons to their phones. Also, according to Mobile Advertising Survey [31], 64% of respondents who have smartphones have made a mobile purchase after seeing a mobile ad. Such apps might be helpful to users who visit large shopping malls with a large number of stores and restaurants, providing information to help them determine which places to visit. Moreover, many users use ad-supported free apps in the app market. Usually, such apps are limited in providing targeted ads, which might eventually make users feel annoyed by irrelevant ads or useless information. If they can provide more relevant ads to users, they might allow the apps to utilize some private user data. Of course, some sensitive users might still be reluctant to use such apps. More in-depth study will be necessary to examine the user acceptance of the visit-pattern-aware mobile ads in shopping malls, and the desired benefits many users expect to obtain from such apps.

**Power consumption:** Power consumption for accelerometer sensing and WiFi scans is an important and practical issue, but we did not address the power consumption issue in this paper. The main focus of the paper was to propose and build a framework to support targeted mobile ads in shopping malls by utilizing place visit patterns. Accordingly, we presented the initial architecture of such a framework and methods to sense place visit patterns (e.g., store entrance and exit, expected visit places). Currently, we design the VisitSense framework to work in an intermittent fashion rather than in a continuous manner (e.g., 24 × 7); it needs to run only when users visit shopping malls. Of course, if users stay at a mall for a long time, power consumption could increase considerably. To address the power consumption issue, diverse approaches, e.g., adaptive duty cycling, hierarchical sensing, could be adopted. We plan to examine the detailed power consumption of the VisitSense framework and to develop efficient visit detection methods in future work.

**Group visit patterns and ads for groups:** In the current study, we did not explicitly classify visitors into groups and examine visit patterns in a shopping mall depending on the type of groups of visitors. However, many shopping mall visitors are likely to be in a group, e.g., a couple or a family. It will be meaningful to analyze group visit patterns and provide different ads targeting a specific group, e.g., a young couple or a family with kids. For example, when a man visits a shopping mall together with his girlfriend, he might visit fashion shops and jewerly shops that he hardly visits with his male friends. Considering such a difference would be desirable for effective targeting. To support such a feature, it is necessary to first determine if a user is a single visitor or she/he is in a group and travels together with others in a shopping mall [32]. Furthermore, VisitSense needs to incorporate diverse group visitor patterns depending on different types of groups, e.g., couple groups, family groups, or friends groups. It will be an interesting future research topic to address such an issue.

**Comprehensive user study:** While the initial result we obtained is positive, the current study is prelimiary and limited in several aspects. First, the participants did not include diverse demographics to represent shopping mall visitors. Second, the number of participatns was small. Third, the period of study was limited; it will be necessary to examine longer term usage of two different advertisements approaches. Conducting a more comprehensive user study will be important work in the future.

## 7. Conclusions

In this paper, we introduced VisitSense, a novel smartphone framework that detects and predicts a smartphone user’s place visits for enabling targeted mobile advertising in shopping malls. Investigating the problems and solutions in a real-world shopping mall, we learned several valuable lessons. First, we observed that, for detecting place visits, the change-based approach was more robust in the noisy radio ambience than the stability-based approach. Second, we identified that using the causality-based approach was intuitive and practical for predicting a user’s next visits. Third, we confirmed that visit-pattern-aware mobile advertising could be more useful for customers and more cost-effective for advertisers.
